# Effects of Stable Vagus Nerve Stimulation Efficacy on Autistic Behaviors in Ten Pediatric Patients With Drug Resistant Epilepsy: An Observational Study

**DOI:** 10.3389/fped.2022.846301

**Published:** 2022-03-02

**Authors:** Zhiyan Wang, Xing Yuan, Qian Zhang, Jialun Wen, Tungyang Cheng, Xiaoya Qin, Taoyun Ji, Xiaomei Shu, Yuwu Jiang, Jianxiang Liao, Hongwei Hao, Luming Li, Ye Wu

**Affiliations:** ^1^National Engineering Laboratory for Neuromodulation, School of Aerospace Engineering, Tsinghua University, Beijing, China; ^2^Department of Pediatrics, Affiliated Hospital of Zunyi Medical University, Zunyi, China; ^3^Department of Pediatrics, Peking University First Hospital, Beijing, China; ^4^Department of Neurology, Shenzhen Children's Hospital, Shenzhen, China; ^5^Precision Medicine & Healthcare Research Center, Tsinghua-Berkeley Shenzhen Institute, Tsinghua University, Shenzhen, China; ^6^IDG/McGovern Institute for Brain Research at Tsinghua University, Beijing, China; ^7^Institute of Epilepsy, Beijing Institute for Brain Disorders, Beijing, China

**Keywords:** vagus nerve stimulation, autistic behavior, pediatric patients, drug resistant epilepsy, comorbidity

## Abstract

Vagus nerve stimulation (VNS) is a safe and effective therapy for pediatric patients with drug-resistant epilepsy (DRE). However, in children with DRE, the effects of VNS on autistic behaviors remain controversial. We retrospectively collected data from 10 children with DRE who underwent VNS implantation and regular parameter regulation in three pediatric epilepsy centers, and completed the behavioral assessments, including the autistic behavior checklist and the child behavior checklist, at follow-ups 1 (mean 2.16 years) and 2 (mean 2.98 years). The 10 children maintained stable seizure control between the two follow-ups. Their autistic behaviors, especially in language, social and self-help, were reduced at follow-up 2 compared to follow-up 1 (*p* = 0.01, *p* = 0.01, respectively). Moreover, these improvements were not associated with their seizure control, whether it was positive or negative. These results suggested that the VNS had a positive effect on autistic behaviors, which provided a preliminary clinical basis that VNS may benefit to younger children with DRE comorbidity autism spectrum disorder (ASD).

## Introduction

Autism spectrum disorder (ASD) is a group of neurodevelopmental dysfunctions characterized by impaired social communication and restricted, repetitive patterns of behavior, interest, or activities (1, 2). Standardized screening for ASD is recommended at 18 and 24 months of age in primary care, but diagnosis later than 6 years of age is reported in one-third to half of children (3). Epilepsy is another common neurologic disorder in children (4). The rate of seizures among people with ASD in clinically ascertained samples has been reported to as high as 46% (5). Vagus nerve stimulation (VNS) is a widely used therapy for patients over 4 years old with drug resistant epilepsy (DRE) (6–8). Children candidate for VNS are at higher risk of behavioral comorbidities or other chronic illnesses, including ASD, compared with those in the general population (9–12).

Vagus nerve is a key component of regulating autonomic nervous system, social emotional function and adaptive behaviors (13). Numerous studies have reported that VNS remarkably improves the quality of life in children with DRE by improving alertness, communication, independence, memory, mood, and sleep (14–19). However, the effects of VNS on autistic behavior in children with DRE is inconsistent. Some case reports supported positive improvements with VNS on autistic behavior in children with DRE and comorbid ASD (20, 21), while other studies have reported the opposite effects (22, 23). Although several studies have observed that the effect of VNS on autistic behavior are independent of seizure control (21–23), within 2 years of VNS implantation, the proportion of pediatric DRE responders (≥50% reduction in seizure frequency) gradually increases from 20 to 60% (24–27). Subsequently, the efficacy of VNS tends to stabilize (27–29). Whether the benefits of autistic behavior improvements are due to VNS or seizure control is still unclear.

Here, we assessed the effects of VNS on autistic behaviors between two follow-ups in ten children with DRE, who achieved stable seizure control. This was the first study to observe the effects of VNS on autistic behavior in children with severe epilepsy at stable seizure control.

## Materials and Methods

### Subjects

Forty children with DRE from three pediatric epilepsy centers (First Hospital of Peking University, Affiliated Hospital of Zunyi Medical University and Shenzhen Children's Hospital) underwent VNS implantation (PINS Medical model G112, Beijing, China) between October 2017 and February 2018. All patients were asked to maintain accurate seizure frequency histories and regular follow-up after VNS implantation. The exclusion criteria were children without regular programming or follow-up. For the present study, 30 children were excluded because they could not come to the hospital due to COVID-19 management and control in China. Therefore, the final sample was 10.

The 10 children included eight boys and two girls. Before VNS implantation, the mean age of these 10 children at epilepsy onset was 0.7 years (range: 0–2.8 years), and the mean duration of epilepsy was 3.9 years (range: 2.6–5.2 years). Two children had experienced epilepsy surgery. The seizure type, etiology, epilepsy syndrome and antiepileptic drugs taken were listed in Table 1.

**Table 1:** Details of the 10 children implanted with VNS.

**Case**	**Age**	**Sex**	**Surgery**	**Antiseizure medications**	**Age at epilepsy onset (year)**	**Duration of epilepsy**	**Seizure type**	**Etiology**	**Epilepsy syndrome**
1	4.1	M	Yes	VPA, LEV, LTG	0.3	3.8	Generalized, Spasm	Structural	IS
2	5.2	F	Yes	VPA, TPM, OXC	0	5.2	Focal	Structural	No
3	4.7	M	No	VPA, LTG, clonazepam	0.5	4.2	Focal	Genetic	No
4	4.4	F	No	VPA, Clobazam	0.4	4.0	Generalized	Unknown	IS
5	4.9	M	No	VPA, LEV, clonazepam	0.2	4.7	Generalized, focal	Genetic	Dravet
6	3.6	M	No	LEV, OXC	0.3	3.3	Generalized, focal	Unknown	GTCS
7	4.7	M	No	VPA, OXC, clonazepam	0	4.7	Generalized, focal, spasm	Structural	IS
8	5.2	M	No	VPA, TPM, OXC	2.2	3.0	Focal	Unknown	No
9	3.6	M	No	VPA, Clobazam	0.5	3.1	Generalized, focal, spasm	Structural	IS
10	5.4	M	No	VPA, OXC	2.8	2.6	Generalized	Structural	No

*IS, infantile spasms; GTCS, generalized tonic–chronic seizures; LEV, levetiracetam; VPA, valproic acid; OXC, oxcarbazepine; LTG, lamotrigine; TPM, topiramate*.

The 10 children were implanted with VNS at a mean age of 4.6 years (range: 3.6–5.4 years). After implantation, the VNS parameters were quickly adjusted to individual optimal stimulation current, and the frequency and duty cycle were 30Hz and 30s on, 5 min off, respectively. During their follow-ups, all the VNS parameters were adjusted based on each child's seizure control. The seizure frequency and VNS stimulation parameters at follow-up 1 (2.16 ± 0.17 years) and follow-up 2 (2.98 ± 0.25 years) are shown in Table 2.

**Table 2:** Seizure, VNS parameters and antiseizure medications in 10 pediatric patients with DRE after VNS stimulation.

**Case**	**Seizure frequency times/month** **(% seizure reduction)**			**VNS parameters (Current mA/Width** **μs/Frequency Hz/On-Off (s-min)**		**Antiseizure medications**	
	**Before VNS implantation**	**Follow-up 1**	**Follow-up 2**	**Follow-up 1**	**Follow-up 2**	**Follow-up 1**	**Follow-up 2**
1	900	120 (86.67)	270 (70.00)	0.5/500/30/30–1.8	0.5/500/30/30–1.1	VPA, LTG, Lacosamide	VPA, LTG, Lacosamide
2	240	225 (6.25)	165 (31.25)	1.3/500/30/30–3	2.0/500/30/30–3	VPA, TPM, OXC	VPA, TPM, OXC
3	30	135 (−350.00)	450 (−1,400)	1.5/500/30/30–1.1	1.3/500/30/30–1.1	VPA, Clobazam	VPA, LTG, Clobazam, Lacosamide
4	90	122 (−35.56)	120 (−25.00)	1.4/500/30/30–5	1.0/500/30/30–5	LEV, VPA	VPA, Clobazam, Perampanel
5	63	28 (55.56)	6 (90.48)	1.8/500/30/30–3	1.9/500/30/30–5	LEV, OXC	VPA, LEV, Clonazepam
6	10	2.5 (75.00)	2.5 (75.00)	1.0/500/30/30–5	1.0/500/30/30–5	VPA, LTG	LEV, OXC
7	300	61 (79.67)	140 (53.33)	1.6/500/30/30–5	1.6/500/30/30–5	VPA, OXC, TPM	VPA, OXC, TPM
8	45	0 (100.00)	0 (100.00)	1.1/500/30/30–5	1.1/500/30/30–5	VPA, TPM	VPA, LTG
9	300	135 (55.00)	210 (30.00)	1.1/500/30/30–5	1.0/500/30/30–5	VPA	VPA, TPM
10	180	0 (100.00)	0 (100.00)	0.5/500/30/30–5	0.5/500/30/30–5	—	VPA

% Seizure reduction = (seizure frequency at baseline—seizure frequency at follow-up)/seizure frequency at baseline ^*^ 100%.

*VPA, valproic acid; LTG, lamotrigine; TPM, topiramate; OXC, oxcarbazepine; LEV, levetiracetam*.

### Ethics

The study was approved by the medical ethical committee of the First Hospital of Peking University, the Affiliated Hospital of Zunyi Medical University and the Shenzhen Children's Hospital. Informed consents from the children's parents were obtained for data collection.

### Measures

#### Autistic Behavior Checklist (ABC)

The ABC is a well-established instrument measuring levels of autistic behavior in individuals with severe disabilities. A total score above 67 indicates autism, and a score above 53 indicates “suspected autism” (30). The ABC assessment included five aspects, namely, sensory, relating, body and object use, language and social and self-care.

#### Child Behavior Checklist (CBCL)

The CBCL is one of the most widely used measures in child psychology and a well-validated parent-reported measure of children's emotional and behavioral functioning (31). The CBCL includes 113 items for which parents were asked to assign a score based on a three-level rating scale indicating how true each item was for their child (0 = “Not true”, 1 = “Somewhat or sometimes true”, 2 = “Very true or often true”). The CBCL produces continuous raw scores and t-scores in each domain. The t-scores, which have a uniform mean of 50 and a standard deviation of 10, are normed separately for boys and girls and for younger (ages 4–11) and older (ages 12–18) children based on a nationally representative sample.

### Procedure

The study assessment protocol included direct child and parent interview (ABC) and parent questionnaires (CBCL). The 10 children and their patients completed the behavioral assessments at follow-up 1 and 2. The ABC were administered by experienced pediatric clinicians, and kept same at the two time-points. The pediatric clinicians at the three centers had previously received consistency training and developed the same autistic behavioral evaluation criteria. The ABC and CBCL scores were calculated separately by another clinician.

### Statistical Analysis

**Figure 1:**
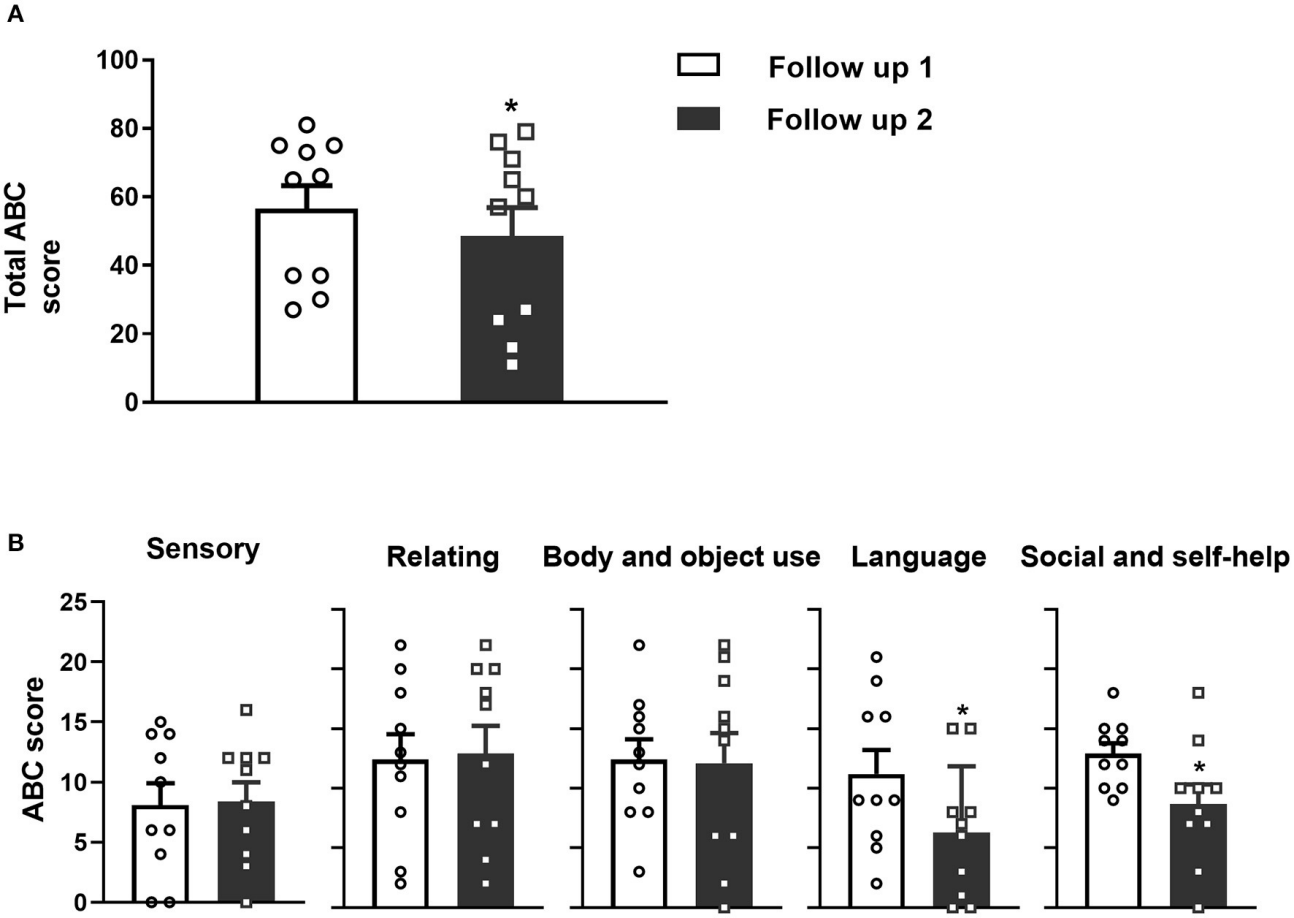
**(A)** Significant decreases in the total ABC scores were observed at follow-up 2 compared to follow-up 1 for the 10 pediatric patients with DRE. **(B)** Five aspects of the specific ABC assessment scores. L and S2 (but not S1, R and B) significantly decreased at follow-up 2 compared to follow-up 1 after VNS implantation. Data are shown as the mean ± SEM, *p < 0.05. S1, Sensory score; R, Relating score; B, Body and object use score; L, Language score; S2, Social and self-help score.

**Figure 2:**
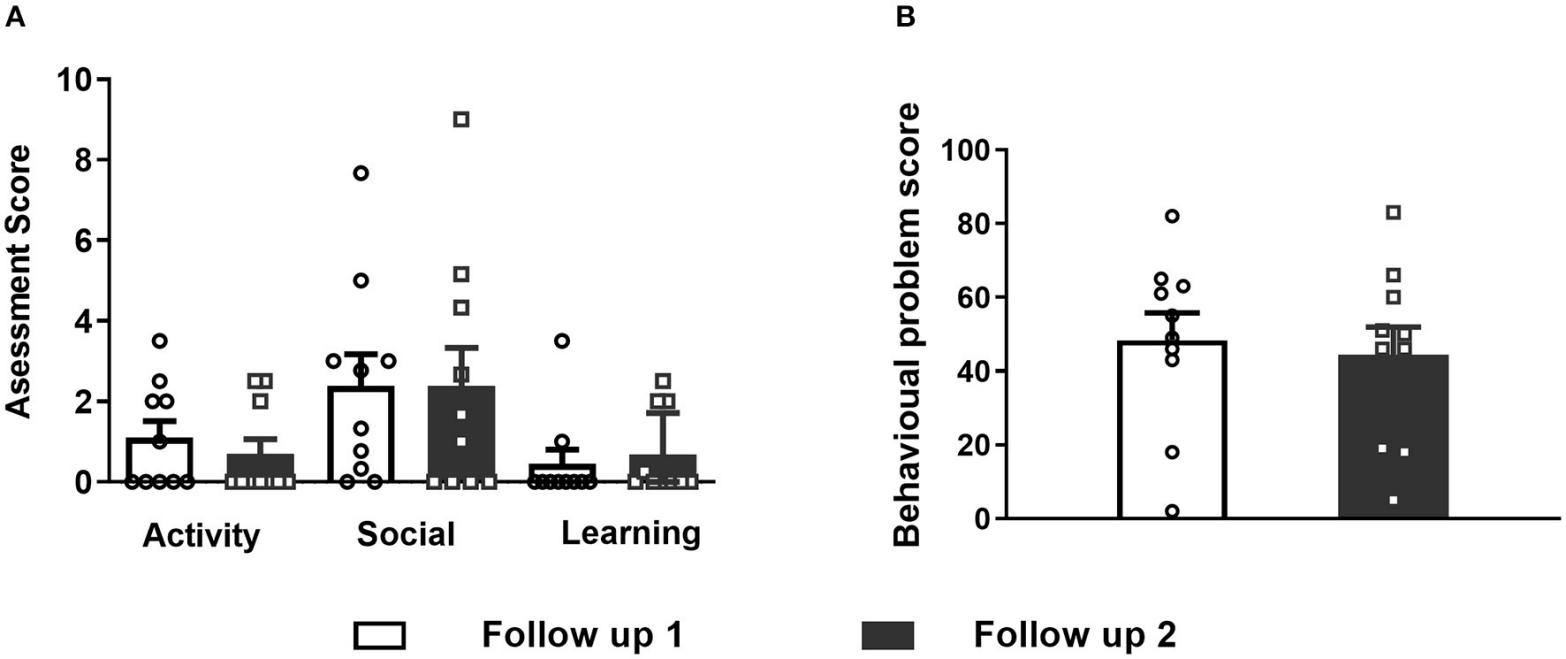
**(A)** Differences were not observed in the activity, social and learning assessment of CBCL between follow-up 1 and 2. **(B)** Total behavioral problem score assessment did not differ between follow-ups 1 and 2 after VNS implantation. Data are shown as the mean ± SEM.

All measurement data are presented as the mean ± standard deviation (SD) in the results description and the mean ± standard error (SEM) in the figures. The ABC and CBCL scores at follow-up 1 and 2 were determined using paired t-tests (shown in Figures 1, 2). The spearman correlation analysis was used between the changes of seizure frequency and autistic behaviors (**Table 4**). All data were analyzed with IBM SPSS (version 20, IBM Corporation, Armonk, New York, USA). Graphs were produced using GraphPad PRISM 8.0. Statistical significance was set at *p* < 0.05.

## Results

### Efficacy of VNS on Seizure

At the first follow-up (follow-up 1), the ten DRE pediatric patients had a mean VNS treatment of 2.16 years (range 1.92–2.58). Seven (cases 1, 5, 6, 7, 8, 9, 10) were responders (seizure frequency reduction ≥50%), two (cases 8 and 10) were seizure-free (no seizure for at least 6 months), and three (case 2, 3, 4) were non-responders (seizure frequency reduction ≤ 50%). At the second follow-up (follow-up 2), the ten children had a mean VNS treatment of 2.98 years (range 2.58–3.5). Six (cases 1, 5, 6, 7, 8, 10) were responders, two (8 and 10) were seizure-free, and four (case 2, 3, 4, 9) were non-responders. The efficacy of VNS was stable in these pediatric patients except for the 9th child. Overall, their seizure control was stable with chronic VNS at the two time-points.

The VNS parameters fine-turned in 6 children between the two follow-ups, including three children who increased their electric current, two children who decreased their electric current and one children who increased the duty cycle. Moreover, seven children changed their antiepileptic medications. The details of the seizure frequency, VNS parameters and antiepileptic medications are shown in Table 2.

### Autistic Behavior Assessment

The total ABC scores of the 10 children at follow-up 1 were 56.6 ± 21.2 (range 27 to 81), and at follow-up 2, the scores decreased to 48.6 ± 26.2 (range 11 to 79). The decrease in ABC scores between follow-ups 1 and 2 was significant (Figure 1A, paired *t*-test, *p* = 0.02). Specifically, four (cases 6, 7, 9, and 10) patients were diagnosed with “autism” according to the total ABC score and two (cases 2 and 5) patients were diagnosed with “suspected autism”. At follow-up 2, three (cases 2, 7 and 10) were diagnosed with “autism” according to the ABC score and three (cases 5, 6, 9) were diagnosed with “suspected autism”. The detailed ABC scores are shown in Table 3.

**Table 3:** ABC assessments after VNS implantation in 10 pediatric patients with DRE.

**Case**	**Follow-up 1**						**Follow-up 2**					
	**T**	**S^**1**^**	**R**	**B**	**L**	**S^**2**^**	**T**	**S^**1**^**	**R**	**B**	**L**	**S^**2**^**
1	37	0	8	8	9	12	11	0	4	0	0	7
2	66	6	22	17	9	12	71	12	20	19	6	14
3	27	0	3	12	2	10	16	3	7	2	1	3
4	37	6	12	3	6	10	27	6	7	14	0	0
5	65	14	13	8	16	18	57	8	18	6	15	10
6	75	12	15	15	19	14	60	11	12	22	7	8
7	73	14	20	16	9	14	76	12	22	16	8	18
8	30	4	2	10	5	9	24	4	2	6	3	7
9	75	15	11	13	21	15	65	12	20	15	8	10
10	81	10	18	22	16	15	79	16	17	21	15	10

1 *T, total score; S, Sensory score; R, Relating score; B, Body and object use score; L, Language score;*

2 *S, Social and self-help score*.

Furthermore, the language scores were 11.2 ± 6.39 (range 2 to 21) at follow-up 1 and 6.35 ± 0.54 (range 0 to 15) at follow-up 2. The self-help scores were 12.9 ± 2.81 (range 9–18) at follow-up 1 and 8.7 ± 5.10 (range 0 to 18) at follow-up 2. Both the language and self-help scores were significantly decreased between follow-up 1 and 2 (Figure 1B, paired *t*-test, *p* = 0.01 and *p* = 0.01, respectively). However, the sensibility, communication and movement scores changed little.

Besides, some children have significant improvements in their autistic behaviors between the two follow ups. For example, case 6 could understand simple instructions, liked to play with his sister and expressed thanks when receiving help. Case 10 increased his interests, such as reading, cooking, writing, playing basketball and cycling.

### CBCL Assessment

Activity, social status and learning did not differ between follow-ups 1 and 2 (Fig 2A). The total behavioral problem score was 48.4 ± 23.41 (range 2 to 82) at follow-up 1 and 44.4 ± 23.98 (range 5–83) at follow-up 2, and these values were not significantly different from the total behavioral problem score (Figure 2B, paired *t*-test, *p* = 0.59).

### Correlation Analysis

Further, to determine whether the improvements of autistic behaviors were associated with seizure control, we analyzed the correlations between the change of seizure frequency and the change of total ABC, language and social and self-help scores between two time points. Results showed that there is no significant correlations between the seizure control and autistic behavior improvements (Table 4).

**Table 4:** Correlations between the change of seizure frequency and the change of autistic behaviors.

		**Change of total ABC score**	**Change of language score**	**Change of social and self-help score**
Change of	r	0.33	0.22	−0.17
seizure frequency	*p* value	0.34	0.54	0.64

## Discussion

Previous studies indicated that the responder rate of VNS for DRE was similar between adults and children (28, 32). Fang et al. recently reported that the response rates of 213 children with DRE were 57.1, 69.2, and 70.7% at 12, 18, and 24 months, respectively (25). Muthiah et al. also reported that the overall response to VNS therapy of 59 patients aged 4–6 years in a single center at 1, 2 and 4 years after VNS implantation was 55, 60, and 52%, respectively (27). These studies showed that the efficacy of VNS gradually increased and was basically stable after 2 years. In our study, the median age of our children at VNS surgery was 4.6 years, and the age ranged from 3.6 to−5.4, which was relatively concentrated at the younger age of children. Seven children (70%) became responders at follow-up 1 (mean 2.16 years) and six (60%) were responders at follow-up 2 (mean 2.98 years). Among the responders, six were stable from follow-up 1 to 2, and two children were stable seizure-free. Our results of chronic VNS for 10 DRE children were consistent with previous studies on seizure control.

In our study, a decrease in ABC score was observed after children achieving stable seizure control, and two of four children changed their diagnosis from “autism” to “suspected autism” under stable seizure control, suggesting the possible positive effects of VNS for autistic behaviors. Moreover, significant reduction of autistic behaviors was observed in language, social and self-help, which were consistent with the enhancement of VNS stimulation in neurocognitive function, including executive functions and language (17, 33, 34). Besides, resent studies reported that the transcutaneous auricular VNS (taVNS) has been demonstrated the positive effects on the regulation of mood and visceral state associated with ASD (13, 35, 36). The taVNS was one of the branches of the vagus nerve (23, 37). As stimulating the vagus nerve directly, VNS shared the similar mechanisms with taVNS at activating the brain regions, triggering neuroimmune modulation and producing treatment effects (23, 36, 38–40). However, the effects of VNS for ASD or comorbid disorders of ASD have been not completely proved, which might be its invasive feature of less application.

It was worth mentioning that the improvements of autistic behaviors over time might be associated with the earlier interventions (including educational practices, or developmental therapies, or behavioral interventions) or getting older (41, 42). Actually, our 10 children haven't gone to school and didn't receive training interventions during the two time points. Besides, these DRE children were with seriously developmental delay. Although the effects of interventions and age cannot be ruled out completely, the improvements in autistic behaviors in these ten children might be partly due to VNS therapy.

Moreover, in addition to VNS, the 10 children also received antiseizure medications (ASMs). In seizure treatments, several evidence pointed to valproate, lamotrigine, and levetiracetam as the most effective and tolerable ASMs for epilepsy in individuals with ASD (43). However, no medications are currently proved to treat core autistic symptoms, including the abnormal language development, impairments in reciprocal social interactions, behavioral inflexibility and repetitive and ritualized behaviors. At present, a major consideration in the ASM therapy of children with ASD is its side effects. The first generation ASMs, such as barbiturates, benzodiazepines, carbamazepine, ethosuximide, phenytoin and valproate, have adverse side effects that in some children could result in or exacerbate epilepsy comorbidities (44). Although some of the second and third generation ASMs have fewer adverse effects, it can't completely be ruled out that in any individual child the ASM could contribute to the comorbidity. In our 10 children, the valproate is a commonly used medicine. Under the circumstances, chronic VNS highly likely contributed to the improvements of language and social and self-care in these children.

In addition, the CBCL assessment showed no significant reduction in autistic behaviors in our study. The CBCL was an emotional/behavioral problems scale that completed by parents (45). It had weaknesses, including biases in assessment and voluntary reporting by relatives of the patients (46). More objective measures or professional reporting will be needed to assess the benefits of VNS for autistic behaviors.

There were still several limitations in our study. First, there was a lack of preoperative baseline assessments of autistic behaviors in these children with DRE, which could better elucidate the role of VNS or seizure control on autistic behaviors. Second, it was not enough for these 10 children to assess their autistic behaviors using ABC alone. Thirdly, the sample size was small. In the future, we will design more rigorous clinical trials to confirm the effects of VNS, medications, and age on autistic behaviors.

In all, our study firstly reported that the VNS had positive effects on autistic behaviors in younger DRE patients at stable seizure control, which suggested that younger children with DRE comorbidity ASD may benefit from VNS therapy. Recently, pairing bursts of VNS with specific movements or sensory events has been shown to improve the rehabilitation of stroke, tinnitus, traumatic brain injury, spinal cord injury, and posttraumatic stress disorder (47). Moreover, behavioral therapies have led to significant gains in intelligence, communication, and social skills in a proportion of children with autism (48–50). In the future, new forms of VNS stimulation or VNS combined with behavioral therapy might be potential better approaches for improving the functional outcomes of individuals with DRE comorbidity ASD, even ASD itself.

## Data Availability Statement

The original contributions presented in the study are included in the article/supplementary material, further inquiries can be directed to the corresponding author/s.

## Ethics Statement

The studies involving human participants were reviewed and approved by the Medical Ethical Committee of Affiliated Hospital of Zunyi Medical University, the Medical Ethical Committee of Peking University First Hospital, and the Medical Ethical Committee of Shenzhen Children's Hospital. Written informed consent to participate in this study was provided by the participants' legal guardian/next of kin. Written informed consent was obtained from the minor(s)' legal guardian/next of kin for the publication of any potentially identifiable images or data included in this article.

## Author Contributions

LL and YW contributed to conception and design of the study. XY, QZ, TJ, and JW collected the data. YJ, YW, XS, JL, and HH organized the database. ZW performed the statistical analysis and wrote the first draft of the manuscript. TC and XQ wrote sections of the manuscript. All authors contributed to manuscript revision, read, and approved the submitted version.

## Funding

This work was supported by the National Natural Science Foundation of China (Grant Number 82101549) and Shenzhen International Cooperative Research Project (GJHZ20180930110402104).

## Conflict of Interest

The authors declare that the research was conducted in the absence of any commercial or financial relationships that could be construed as a potential conflict of interest.

## Publisher's Note

All claims expressed in this article are solely those of the authors and do not necessarily represent those of their affiliated organizations, or those of the publisher, the editors and the reviewers. Any product that may be evaluated in this article, or claim that may be made by its manufacturer, is not guaranteed or endorsed by the publisher.
